# Etiology and Clinical Characteristics of Single and Multiple Respiratory Virus Infections Diagnosed in Croatian Children in Two Respiratory Seasons

**DOI:** 10.1155/2016/2168780

**Published:** 2016-08-30

**Authors:** Sunčanica Ljubin-Sternak, Tatjana Marijan, Irena Ivković-Jureković, Jasna Čepin-Bogović, Alenka Gagro, Jasmina Vraneš

**Affiliations:** ^1^Medical Microbiology Department, School of Medicine, University of Zagreb, Zagreb, Croatia; ^2^Clinical Microbiology Department, Teaching Institute of Public Health “Dr. Andrija Stampar”, Zagreb, Croatia; ^3^Department of Pulmonology, Allergy, Immunology and Rheumatology, Children's Hospital Zagreb, Zagreb, Croatia; ^4^Pediatric Department, Faculty of Medicine, University of Osijek, Osijek, Croatia

## Abstract

The aim of this study was to determine the causative agent of acute respiratory infection (ARI) in hospitalized children, as well as investigate the characteristics of ARIs with single and multiple virus detection in two respiratory seasons. In 2010 and 2015, nasopharyngeal and pharyngeal swabs from a total of 134 children, admitted to the hospital due to ARI, were tested using multiplex PCR. Viral etiology was established in 81.3% of the patients. Coinfection with two viruses was diagnosed in 27.6% of the patients, and concurrent detection of three or more viruses was diagnosed in 12.8% of the patients. The most commonly diagnosed virus in both seasons combined was respiratory syncytial virus (RSV) (28.6%), followed by parainfluenza viruses (PIVs) types 1–3 (18.4%), rhinovirus (HRV) (14.3%), human metapneumovirus (10.1%), adenovirus (AdV) (7.1%), influenza viruses types A and B (4.8%), and coronaviruses (4.2%). In 2015, additional pathogens were investigated with the following detection rate: enterovirus (13.2%), bocavirus (HBoV) (10.5%), PIV-4 (2.6%), and parechovirus (1.3%). There were no statistical differences between single and multiple virus infection regarding patients age, localization of infection, and severity of disease (*P* > 0.05). AdV, HRV, HBoV, and PIVs were significantly more often detected in multiple virus infections compared to the other respiratory viruses (*P* < 0.001).

## 1. Introduction

Acute respiratory infections (ARIs) are the most common infections in humans of all ages. Children and infants are one of the most vulnerable groups of the population, and ARIs are the most common cause of children's hospitalization worldwide [1]. Although bacteria, fungi, and parasites can cause ARIs, respiratory viruses cause the majority of infections. Most respiratory virus infections in early childhood are confined to the upper respiratory tract. About one-third of infants develop lower respiratory tract infection (LRTI) [2]. The most common causative viral agents of ARIs in children, respiratory syncytial virus (RSV), human metapneumovirus (HMPV), influenza viruses (Flu), and adenoviruses (AdV), were the subject of intensive research for years; therefore, clinical characteristics and regional epidemiological features of those ARIs in Croatia are well known [3–6]. However, the list of respiratory viruses is growing due to the rapid advance of laboratory diagnostic methods. In the last ten years, newly discovered viruses have been identified including human bocavirus (HBoV), coronaviruses NL63 (HCoV-NL63) and HKU1 (HCoV-HKU1), new enterovirus (HEV), parechovirus (HPeV), and rhinovirus (HRV) strains [7]. Additionally, despite the fact that some of the respiratory viruses have been well known for a long time, particularly parainfluenza type 4 (PIV-4), the technically demanding cultivation methods and unavailability of commercial tests made it difficult to diagnose PIV-4's infection [8, 9]. Infections caused by some of the newly discovered viruses (i.e., HBoV, HCoV-NL63, and HCoV-HKU1) as well as those difficult to cultivate (PIV-4) have not been recorded in the country yet. There are few recent studies from the region providing valuable but still insufficient data regarding regional epidemiology of infections caused by the abovementioned viruses [10, 11]. Furthermore, the issue of multiple respiratory virus detection, which occurred because of high sensitivity of molecular methods, complicates the interpretation of laboratory diagnosis. The aim of this study was to determine the viral etiology for sixteen viruses tested by multiplex PCR method among children with ARI admitted to the hospital in Zagreb region in two respiratory seasons, in order to demonstrate the need for molecular diagnostics introduced in routine practice. Also, we aimed to investigate the characteristics of infections with single and multiple virus detection, especially regarding the type of virus involved and severity of infection.

## 2. Materials and Methods

### 2.1. Patients and Specimens

A total of 134 children admitted to Children's Hospital Zagreb during two winter seasons (January to March) in 2010 and 2015 with symptoms of ARI and suspected for viral etiology (normal or slightly elevated inflammatory markers, i.e., white cell count) were included in the study. Patients were categorized into three groups according to age (<1, 1–3, and ≥4 years of age) and two groups according to the localization of infection in those with upper respiratory tract infection (URTI) and lower respiratory tract infection (LRTI). URTI was defined by symptoms of the common cold, coryza, cough, and hoarseness often accompanied with fever. Clinical syndromes of respiratory catarrh, rhinitis, and/or pharyngitis are included in URTI category. LRTI was defined according to the clinical symptoms of tachypnea, wheeze, severe cough, breathlessness, and respiratory distress accompanied by LRTI signs such as nasal flaring, jugular, intercostal, and thoracic indrawings, rarely cyanosis, and, on auscultation of the chest, wheeze, crackles, crepitations, and inspiratory rhonchi or generally reduced breath sounds [2]. Clinical syndromes of bronchitis, bronchiolitis, and pneumonia were included in LRTI category. To avoid unnecessary X-ray exposure, chest radiographs were taken only for some of the patients to exclude or confirm bacterial pneumonia. Severe disease and acute respiratory distress syndrome (ARDS) were defined with need for oxygen supplementation and/or mechanical ventilation. The patients' underlying conditions data were collected retrospectively from medical charts. The most common underlying diseases were asthma, anamnestic recurrent wheezing episodes, neurological disorders, prematurity, and anemia. Written consent was obtained from the children's parents or caretakers. The study was approved by the Ethic Committee of the Teaching Institute of Public Health “Dr. Andrija Stampar.”

Nasopharyngeal and pharyngeal flocked swabs from each patient were collected, combined, and placed in viral transport medium (UTM*™*, Copan, Italy). Specimens accompanied with demographic data and clinical diagnosis were immediately transported to the Molecular Microbiology Laboratory at the Public Health Institute where they were stored at −80°C until tested. Nasopharyngeal and pharyngeal swabs, blood cultures, and serum for serology were searched to exclude bacterial infection. Patients with samples positive on bacteriology testing were subsequently excluded from the study.

### 2.2. Laboratory Testing

**Figure 1:**
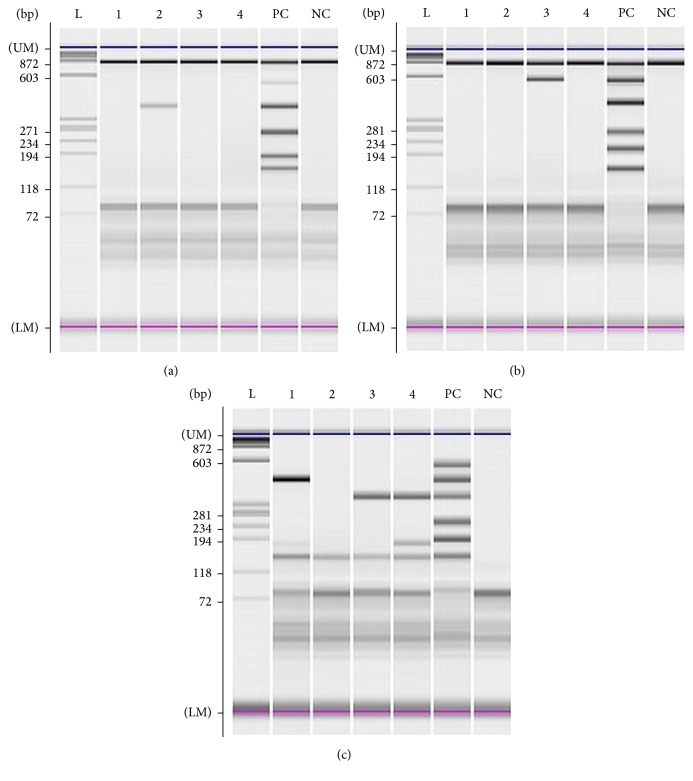
Gel image of electropherogram for (a),  (b), and (c) sets of Seeplex® RV15 OneStep ACE Detection test: (L) ladder; (1) sample negative in sets (a) and (b) and positive for influenza B and enterovirus in set (c); (2) sample positive for human coronavirus 229/NL63 in set (a) and negative in set (b) and set (c); (3) sample negative in set (a), positive for human coronavirus OC43 in set (b), and positive for metapneumovirus in set (c); (4) sample negative in sets (a) and (b) and positive for metapneumovirus and enterovirus in set (c). PC: positive control; set (a) = 859 bp/PCR control, 534 bp/adenovirus, 375 bp/coronavirus 229/NL63, 264 bp/parainfluenza type 2, 189 bp/parainfluenza type 3, and 153 bp/parainfluenza type 1; set (b) = 850 bp/PCR control, 578 bp/coronavirus OC43, 394 bp/rhinovirus, 269 bp/respiratory syncytial virus A, 206 bp/influenza A, and 155 bp/respiratory syncytial virus B; set (c) = 579 bp/bocavirus, 456 bp/influenza B virus, 351 bp/metapneumovirus, 254 bp/parainfluenza virus type 4, 194 bp/enterovirus, and 153 bp/whole process control; NC: negative control.

To isolate viral DNA and RNA from viral transport medium, 200 *μ*L was extracted according to the manufacturer's protocol using QIAamp®MinElute®Virus Spin Kit (Qiagen, Hilden, Germany). Specimens collected in 2010 were tested using multiplex based PCR test for detection of 12 respiratory viruses, and specimens collected in 2015 were tested for 15 respiratory viruses using Seeplex RV12 and Seeplex RV15 detection kit, respectively (Seegene Inc., Seoul, Korea). Briefly, multiplex PCR and cDNA synthesis was performed in one-step reaction using thermal cycler GeneAmp® 9700 PCR System (Applied Biosystems, Foster City, USA) followed by microchip electrophoresis detection on MCE®-202 MultiNA device (Shimadzu, Kyoto, Japan) including software analysis that displays result in form of electropherogram and virtual gel (Figure 1). Parechovirus was detected performing real-time RT-PCR using LightMix® Modular Parechovirus kit (TIB MOLBIOL, GmbH, Berlin, Germany) on LightCycler 480 II Instrument (Roche Diagnostics GmbH, Mannheim, Germany) according to the manufacturer's protocol.

Comparison between groups was performed using the Chi-square test, and statistical analysis was done using STATISTICA 12.7. *P* < 0.05 was considered significant.

## 3. Results

There were 62 patients examined and tested in 2010 and 72 patients in 2015, respectively. Patients were one month to 16 years of age with median age 3 ± 3.34 years in 2010 and 3 ± 3.39 years in 2015. Overall, there were 56 girls (41.8%) and 78 boys (58.2%) with female-to-male ratio of 1 : 1.4 (1 : 1.3 in 2010 and 1 : 1.5 in 2015, resp.). Viral etiology was established in 109 out of 134 (81.3%) patients with ARI (54/62, 87% in 2010; 55/72, 76.4% in 2015, resp.). There were 43 (39.4%) female and 66 (60.6%) male infected children with female-to-male ratio of 1 : 1.5. Infected children's characteristics and characteristics of infection including number of pathogens detected, localization, and severity of disease for each season are presented in Table 1. There were no statistical significant differences between the two investigated seasons regarding the abovementioned categories (Table 1). Average length of hospital stay was 6.2 ± 5.4 days in both seasons.

A single virus was diagnosed in 61.3% (65/109) of the patients, coinfection with two viruses in 27.6% (30/109) of the patients, and concurrent detection of three viruses in 11.0% (12/109) of the patients. There were two cases of concurrent detection of four viruses (1.8%) one in each of the investigated seasons. There were no statistical significant differences between single and multiple virus infection regarding patients age (*P* = 0.0998), localization of infection (*P* = 0.3818), and severity of disease (*P* = 0.5147). However, some of the viruses were significantly more often detected in multiple infection combination than other viruses. These are AdV (*P* = 0.0013), HRV (*P* < 0.0001), PIVs (*P* < 0.0001), and HBoV (*P* = 0.0002).  Table 2 presents the incidence of certain viruses and their representation in single infection and coinfection.

**Figure 2:**
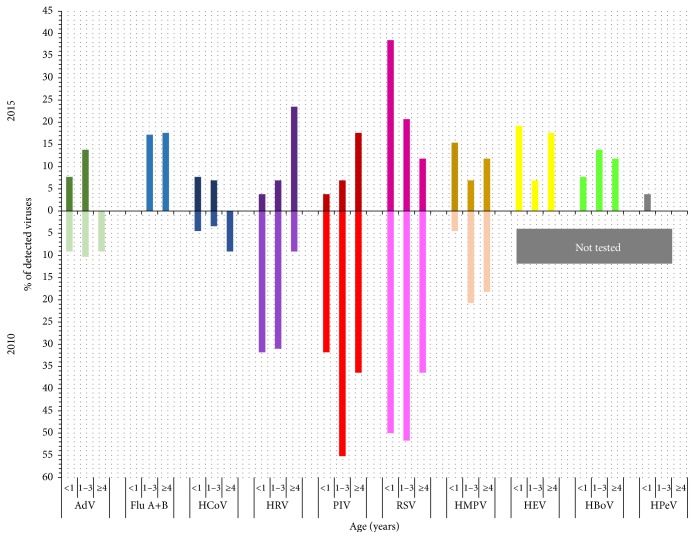
Viral incidence by patient's age and viral type in 2010 and 2015.

The most commonly diagnosed virus in both seasons combined was RSV (28.6%; 48/168), followed by PIVs types 1–3 (PIV-3 12.5%, 21/168; PIV-1 3.6%, 6/168; and PIV-2 2.3%, 4/168), HRV (14.3%, 24/168), HMPV (10.1%, 17/168), AdV (7.1%, 12/168), Flu A+B (4.8%, 8/168), and HCoV (4.2%, 7/168). However, incidence of viruses differs between seasons (Figure 2). There were no Flu viruses detected in 2010 and eight of them were detected in 2015 (*P* = 0.0014). PIVs were significantly more often detected in 2010 than in 2015 (*P* = 0.0001) with the highest frequency of PIV-3 detection in both seasons. Distribution among the types of PIV did not differ between the seasons (*P* = 0.4854).

In 2015, four additional viruses were tested, HEV, HBoV, PIV-4, and HPeV, revealing the following detection rate in 2015: HEV 13.2%, HBoV 10.5%, PIV-4 2.6%, and HPeV 1.3%, respectively.

The highest number of all viral infections was diagnosed in the 1–3-year-old group in both seasons (54.4%, 50/92 in 2010 and 38.2%, 29/76 in 2015) with observed differences in season's incidence of PIV and HRV in relation to the age of the patients (Figure 2). In children below one year of age, higher incidence of HRV and PIV in 2010 compared to 2015 was recorded (*P* = 0.0092 and *P* = 0.0092, resp.), and in children 1–3 years of age higher incidence of PIV in 2010 than in 2015 (*P* = 0.003) was also observed. RSV incidence was the highest in 1–3-year-old children in 2010 in contrast to the highest RSV incidence in <1-year-old group in 2015. However, there were no statistical differences observed comparing RSV incidence between the seasons in relation to patients age (*P* = 0.426 for <1-year-old group and *P* = 0.062 for 1–3-year-old group, resp.). HRV was significantly more often detected in children with URTI (*P* = 0.0082), while RSV was significantly more often detected in children with LRTI (*P* < 0.0001) compared to the other viruses.

## 4. Discussion

Determining ARIs etiology solely based on symptoms, clinical findings, and biochemical tests without adequate laboratory testing is not possible because pathogen-specific clinical symptoms are lacking. Establishment of viral etiology by using traditional methods such as viral cultures is too slow for clinical purposes and technically laborious. Direct immunofluorescence assay (DFA) and rapid antigen test yield results within very few hours, but in many cases they lack sensitivity and specificity and are available for few viruses only [12]. Therefore, the current gold standard (e.g., viral culture or DFA) for detecting conventional respiratory viruses such as Flu, RSV, AdV, and PIVs types 1–3 will be challenged and eventually replaced by the nucleic acid amplification techniques (NAATs), which are not routinely performed in Croatia. Even more, for newly discovered viruses such as HBoV, NAATs were the only available method for laboratory diagnosis until recently [13]. Additionally, recently developed multiplex PCR methods enable testing for many pathogens in parallel in a single analysis, and commercial tests based on clinical syndrome approach are available [14]. Viral etiology in this study was investigated using two multiplex PCR tests: one with a panel of 12 viruses performed in season 2010 and the new variant of the same test with extended spectrum of viruses that included detection of HBoV, PIV-4, and HEV performed in respiratory season 2015. This resulted with detection of HBoV and PIV-4 in the Croatian children population for the first time, thus emphasizing the necessity of using the detection method that will cover all known respiratory viruses in future diagnostic approach. The performance of the used assay always should be considered in interpretation of the result. The characteristics and limitations of the assay used in this study were the relatively low sensitivity of 54.17%, but excellent specificity of 98.41, accuracy of 0.96, and agreement with kappa coefficient of 0.81 when compared to the performance of the Argene/bioMerieux duplex tests used as gold standard [14]. The limitations of this observational study also should be noted. They include relatively small sample size indicating the need for larger studies to reach more accurate epidemiological data and precise sampling approach with strictly determined period of sample collection regarding the onset of ARI that can result with higher virus detection rate.

The most commonly detected virus in both seasons as well as important cause of LRTI was RSV that is in line with the other studies that investigated etiology of ARI in hospitalized children [15–17]. RSV incidence according to the children's ages did not statistically differ between the seasons, but the typical pattern decrease of RSV detection with patient's age was observed in 2015. In 2010, the second and third most commonly detected viruses were PIV-3 and HRV, respectively, while in 2015 detection of RSV was followed by HEV, and both HMPV and HBoV were detected with equal frequencies. PIV-3 was the most prevalent type of all PIVs detected in both seasons, which is in accordance with PIVs type prevalence in other countries [18]. The present study revealed difference between investigated seasons in detection of Flu viruses with no Flu viruses detected in 2010 and eight Flu strains detected in 2015. Detailed retrospective data analysis on influence activity provided by WHO collaboration centre for influenza surveillance in Croatia showed that the 2009/2010 season was characterized by an unusual epidemiological pattern. Peak of incidence was recorded in autumn and at the end of activity in December of 2009 due to the emergence of a pandemic strain, and there were no reported cases at the beginning of 2010. On the contrary, high activity of influenza was recorded in the beginning of 2015 with influenza peak incidence in February [19]. In this study, HMPV was detected in 10.1% in both seasons combined, with no difference between the seasons, which is in line with published HMPV incidence ranging between 7% and 19% in both hospitalized children and outpatients with ARI [3, 7]. Although previous studies suggested that HMPV has been more frequently detected in children with LRTI [6], this study did not find a difference between HMPV detection rates according to the localization of infection. Molecular detection of HRV and HEV revealed the true significance of these viruses in the etiology of ARI. It seems that HRV and respiratory HEV are leading causes of upper respiratory tract infections, but molecular diagnostic techniques have revealed the presence of HRV in the lower respiratory tract as well, and its role in lower airway diseases is increasingly reported [20]. Results of the present study showed that HRV was more frequently detected together with other respiratory viruses, as well as in children presenting with URTI. There are many types of HRV and HEV, which disable the use of rapid test such as DFA, and cultivation is a method of choice only for those that are easily grown in cell culture, which is not characteristic for many respiratory types of HEVs. HEVs cause many clinical syndromes. Types of HEVs that cause aseptic meningitis and febrile illness with or without exanthema most commonly infect children of preschool and school age [21]. Half the number of all detected HEVs in this study was recorded in children under 1 year of age, which indicated that infections with respiratory types of HEVs appear in early childhood. In order to investigate as much as possible viral causes of ARI in the present study, monoplex real-time PCR for HPeV was performed in the season 2015, and one infection was diagnosed in a child <1 year of age. Though frequently detected in retrospective studies and usually associated with gastrointestinal and respiratory symptoms, the diagnostics of HPeVs are still not included in routine screening for acute diarrhoea or respiratory syndromes. Based on the previous experience and reports [22], they should be included in differential diagnosis of respiratory infection even if only in small children.

This study revealed high coinfection detection rate of 40.4% infections in both seasons combined. Coinfection rates in two recently published studies from China and Brazil that search for 18 and 13 respiratory viruses in nasopharyngeal and pharyngeal secretions of patients with ARI were 18% and 65%, respectively [17, 23]. There were no significant differences between single and multiple virus infection regarding patient's age, localization of infection, and severity of disease. Recently published meta-analysis also demonstrated that viral coinfection did not increase severity in all outcomes assessed (i.e., need of hospitalization, length of stay, need of supplemental oxygen, intensive care, and mechanical ventilation) [24]. However, the present study showed that AdV, HRV, PIVs, and HBoV were significantly more often detected in multiple infection combination than the other viruses. It seems to be that coinfections are related to the prolonged period of viral persistence in the mucosa of the respiratory tract. In particular, HBoV-1 primary infection is associated with mild respiratory illness with subsequent prolonged detection of HBoV-1 DNA for up to a year [25]. Broccolo et al. also advocate the hypothesis in which virus persistence plays a role in the high frequency of coinfections with proper pathogens of URTI and LRTI [26]. The importance of recognizing the true respiratory pathogen in multiple viral respiratory infection was also emphasized in a meta-analysis published by Shi et al. It showed strong evidence for causal attribution of RSV, Flu, PIV, and HMPV in young children presenting with LRTI compared to asymptomatic or healthy children, with less strong evidence for HRV, but no significant difference in the detection of AdV, BoV, or CoV in cases and controls [27]. Therefore, detected specific sequences of nucleic acid of HMPV, Flu, PIVs, or RSV in respiratory specimens enable conclusions about the causative pathogen, but for the evaluation of the detection of other pathogens (i.e., HBoV, HCoV, HRV, and AdV) a clinical evaluation is needed in order to distinguish acute infections from subclinical events with nucleic acid persistence. Several studies indicated that symptomatic infections are associated with higher viral load [28, 29]. Although the multiplex PCR test used in this study is declared as a qualitative test, there were differences in the intensity of the virus specific bands observed in some coinfections, giving the impression that the virus whose detection has resulted in lower intensity band was actually present in smaller amounts in the sample (Figure 1). Therefore, the measurement of viral load in respiratory specimen could be possible progress in identifying the true respiratory pathogen [30].

## 5. Conclusion

In conclusion, RSV remains the most common but not the only pathogen among children presenting with ARI, especially in those under 3 years of age. Multiplex PCR enables detection of many viruses, and all possible pathogens should be included in laboratory diagnosis of ARI. Multiple virus detection still represents the challenge in the interpretation of the result. Quantitative molecular diagnostics may contribute to the clarification between coinfection and codetection of respiratory viral pathogens.

## Figures and Tables

**Table 1:** Characteristics of patients with laboratory confirmed viral respiratory infection in 2010 (*N* = 54) and 2015 (*N* = 55).

	2010	2015	*P* value
	*N* (%)	*N* (%)	
Gender			
Male	31 (57.4)	35 (63.6)	0.5577
Female	23 (42.6)	20 (36.4)	
Age (years)			
<1	19 (35.2)	22 (40.0)	0.3698
1–3	26 (48.1)	20 (36.4)	
≥4	9 (16.7)	13 (23.6)	
Underlying disease			
Yes	16 (29.6)	24 (43.6)	0.1128
No	38 (70.4)	31 (56.4)	
Hospitalization			
Yes	41 (75.9)	45 (81.8)	0.4818
No	13 (24.1)	10 (18.2)	
Number of pathogens detected			
Single	27 (50.0)	38 (69.1)	0.0516
Multiple	27 (50.0)	17 (30.9)	
Localization			
URTI	20 (37.0)	22 (40.0)	0.5955
LRTI	10 (18.6)	14 (25.4)	
URTI + LRTI	24 (44.4)	19 (34.6)	
Severity of disease			
Mild/moderate	50 (92.6)	49 (89.1)	0.5071
Severe/ARDS	4 (7.4)	6 (10.9)	

**Table 2:** Viral etiology and coinfections identified in 109 infected children in two respiratory seasons.

	2010^**∗**^	2015^**∗∗**^	Total
	*N* (single + coinfection)	*N* (single + coinfection)	*N* (single + coinfection)
AdV	6 (1 + 5)	6 (1 + 5)	12 (2 + 10)
Flu A	0 (0 + 0)	3 (1 + 2)	3 (1 + 2)
Flu B	0 (0 + 0)	5 (4 + 1)	5 (4 + 1)
HCoV 229E/NL63	1 (1 + 0)	2 (1 + 1)	3 (2 + 1)
HCoV OC43/HKU1	2 (0 + 2)	2 (1 + 1)	4 (1 + 3)
HRV	17 (2 + 15)	7 (4 + 3)	24 (6 + 18)
PIV 1	6 (1 + 5)	0 (0 + 0)	6 (1 + 5)
PIV 2	3 (1 + 2)	1 (0 + 1)	4 (1 + 3)
PIV 3	18 (7 + 11)	3 (0 + 3)	21 (7 + 14)
PIV 4	NA	2 (0 + 2)	2 (0 + 2)
RSV A	4 (0 + 4)	8 (6 + 2)	12 (6 + 6)
RSV B	26 (11 + 15)	10 (7 + 3)	36 (18 + 18)
HMPV	9 (3 + 6)	8 (6 + 2)	17 (9 + 8)
HEV	NA	10 (5 + 5)	10 (5 + 5)
HBoV	NA	8 (1 + 7)	8 (1 + 7)
HPeV^*∗∗∗*^	NA	1 (1 + 0)	1 (1 + 0)

Total	92 (27 + 27 cases)	76 (38 + 17 cases)	168 (65 + 44 cases)

NA: not applicable.

^*∗*^Diagnosed by RV12 multiplex PCR test.

^*∗∗*^Diagnosed by RV15 multiplex PCR test.

^*∗∗∗*^Diagnosed by real-time PCR.
